# Supplementary material for: Creating Majorana modes from segmented Fermi surface
Source: Nat Commun. 2021 Jan 25;12:577. doi: 10.1038/s41467-020-20690-3 (PMC7835351; doi:10.1038/s41467-020-20690-3)
Supplement: Supplementary file 1 — Supplementary Information [file 41467_2020_20690_MOESM1_ESM.pdf]

Supplementary Material for "Creating Majorana modes from segmented Fermi surface"

Michał Papaj and Liang Fu
Department of Physics, Massachusetts Institute of Technology, Cambridge, Massachusetts 02139, USA

SUPPLEMENTARY NOTE 1: DERIVATION OF THE PROJECTED HAMILTONIAN

To simplify the analysis of the problem of a proximitized Dirac surface state in in-plane magnetic field, we first notice that the chemical potential $\mu \gg \Delta, B_x$ is the largest energy scale of the problem. This allows us to project the original Hamiltonian (1) of the main text to just the electron and hole component of the band closest to Fermi energy. To do so, we first diagonalize the problem in the absence of superconductivity ($\Delta = 0$), which yields the following eigenenergies and spinors:

$$E_{1,\pm} = \pm|k_x v + i(B_x - k_y v)| - \mu, \qquad \psi_{1,\pm} = \frac{1}{\sqrt{2}}(\mp i e^{i\alpha_-}, 1, 0, 0)^T \tag{1}$$

$$E_{2,\pm} = \pm|k_x v + i(B_x + k_y v)| + \mu, \qquad \psi_{2,\pm} = \frac{1}{\sqrt{2}}(\pm i e^{-i\alpha_+}, 1, 0, 0)^T \tag{2}$$

where we define $\alpha_\pm = \mathrm{Arg}\,(k_x v + i(B_x \pm k_y v))$. We can now express the full Hamiltonian with superconductivity from Eq. (1) of the main text in the basis of these four states. However, for large μ in each of the pairs of states above there is only one ($\psi_{1,+}$ and $\psi_{2,-}$) whose energy is close to μ and thus is relevant to the low energy properties of the system. Moreover, these states are only weakly coupled to the remaining pair by a term of magnitude $\sim \Delta B_x/\mu$. Therefore, we can neglect the remaining states and focus only on the two low energy states, obtaining in this way the projected Hamiltonian (2) of the main text:

$$H_p = \begin{pmatrix} kv - \mu - B_x k_y/k & \Delta \\ \Delta & -kv + \mu - B_x k_y/k \end{pmatrix}$$

with the following eigenvalues:

$$E_\pm = \pm\sqrt{(kv-\mu)^2 + \Delta^2} - B_x \frac{k_y}{k} \tag{3}$$

These eigenvalues are presented in Fig. 2(a) of the main text for two values of magnetic field. When $B_x > \Delta$, the zero energy contours for these bands form the segmented Fermi surface as shown in Fig. 2(b) of the main text.

SUPPLEMENTARY NOTE 2: SCATTERING MATRIX APPROACH CALCULATION

Using the low energy Hamiltonian we can now obtain the subgap quasiparticle modes underneath the magnetic strip. We consider a strip infinite in the x direction with translational invariance, which allows us to parametrize the states by their energy and longitudinal momentum k_x. Since the projected basis is momentum-dependent, it is useful to transform it back to the original basis to solve the scattering problem in the y direction. When $B_x > \Delta$, there are four states on the segmented Fermi surface for given k_x, two electron-dominated and two hole-dominated, that are moving in the opposite directions along the y axis. These states, expressed in the initial basis of the original Hamiltonian, are approximately given by:

$$\psi_{e,+} = \frac{1}{2}\left(-ie^{i\alpha_- - i\beta_+}, e^{-i\beta_+}, -ie^{-i\alpha_+}, 1\right)^T e^{ik_x x + ik_{e,+} y} \tag{4a}$$

$$\psi_{e,-} = \frac{1}{2}\left(-ie^{i\alpha_+ + i\beta_-}, e^{i\beta_-}, -ie^{-i\alpha_-}, 1\right)^T e^{ik_x x + ik_{e,-} y} \tag{4b}$$

$$\psi_{h,+} = \frac{1}{2}\left(-ie^{i\alpha_- + i\beta_+}, e^{i\beta_+}, -ie^{-i\alpha_+}, 1\right)^T e^{ik_x x + ik_{h,+} y} \tag{4c}$$

$$\psi_{h,-} = \frac{1}{2}\left(-ie^{i\alpha_+ - i\beta_-}, e^{-i\beta_-}, -ie^{-i\alpha_-}, 1\right)^T e^{ik_x x + ik_{h,-} y} \tag{4d}$$

with $\beta_\pm = -\arccos\left((\epsilon \pm B_x k_0 v/\mu)/\Delta\right)$ and $k_{e/h,\pm}$ given in the main text. Each of these wavefunctions carries a quasiparticle current, which for the Hamiltonian under consideration is given by:

$$\mathbf{j}_{\rm qp} = 2{\rm Im}\psi^\dagger i(\tau_z s_y, -\tau_z s_x)\psi \tag{5}$$

As we want to use the wavefunctions of Supplementary Eq. (4) as a basis for the scattering matrices, we have to normalize them so that they carry the same current in the direction perpendicular to the interface at the boundary of the narrow strip:

$$\tilde{\psi}_{e/h,\pm} = \frac{\psi_{e/h,\pm}}{N_{e/h,\pm}}, \qquad N_{e/h,\pm} = \sqrt{|j_{\rm qp,y}(\psi_{e/h,\pm})|} \tag{6}$$

Inside of the surrounding superconductor there will be also four possible solutions, but since $B_x = 0$ in that region, for ϵ within the gap Δ the solutions will be exponentially decaying in either $+y$ or $-y$ direction. These wavefunctions are:

$$\psi_{SC1} = \frac{1}{2}\left(-ie^{i\alpha_0+i\beta_0}, e^{i\beta_0}, -ie^{i\alpha_0}, 1\right)^T e^{ik_x x-\kappa y} \tag{7a}$$
$$\psi_{SC2} = \frac{1}{2}\left(-ie^{-i\alpha_0-i\beta_0}, e^{-i\beta_0}, -ie^{-i\alpha_0}, 1\right)^T e^{ik_x x-\kappa y} \tag{7b}$$
$$\psi_{SC3} = \frac{1}{2}\left(-ie^{-i\alpha_0+i\beta_0}, e^{i\beta_0}, -ie^{-i\alpha_0}, 1\right)^T e^{ik_x x+\kappa y} \tag{7c}$$
$$\psi_{SC4} = \frac{1}{2}\left(-ie^{i\alpha_0-i\beta_0}, e^{-i\beta_0}, -ie^{i\alpha_0}, 1\right)^T e^{ik_x x+\kappa y} \tag{7d}$$

where $\beta_0 = \arccos\epsilon/\Delta$, $\alpha_0 = {\rm Arg}(k_x + ik_y)$ and $\kappa = \frac{k_F}{k_0}\sqrt{\epsilon^2/v^2 - \Delta^2/v^2}$.

Equipped with the wavefunctions in both regions we can now derive the normal and Andreev reflection coefficients at the two interfaces at $y = \pm W/2$. To this we solve the set of equations:

$$\tilde{\psi}_{e,+} + r_{N1}\tilde{\psi}_{e,-} + r_{A1}\tilde{\psi}_{h,+} = a_1\psi_{SC1} + b_1\psi_{SC2} \tag{8a}$$
$$\tilde{\psi}_{h,-} + r'_{N1}\tilde{\psi}_{h,+} + r'_{A1}\tilde{\psi}_{e,-} = a'_1\psi_{SC1} + b'_1\psi_{SC2} \tag{8b}$$
$$\tilde{\psi}_{e,-} + r_{N2}\tilde{\psi}_{e,+} + r_{A2}\tilde{\psi}_{h,-} = a_2\psi_{SC3} + b_2\psi_{SC4} \tag{8c}$$
$$\tilde{\psi}_{h,+} + r'_{N2}\tilde{\psi}_{h,-} + r'_{A2}\tilde{\psi}_{e,+} = a'_2\psi_{SC3} + b'_2\psi_{SC4} \tag{8d}$$

We solve these equations for the complex reflection coefficients r_N (normal reflection) and r_A (Andreev reflection) and for clarity extract the phases acquired during the propagation across the strip region into the transmission matrices as indicated in the main text. The reflection coefficients at both interfaces can then be arranged into two scattering matrices $S_{\pm W/2}$ at both boundaries of the narrow strip region:

$$S_{W/2} = \begin{pmatrix} r_{N1} & r'_{A1} \\ r_{A1} & r'_{N1} \end{pmatrix} \qquad S_{-W/2} = \begin{pmatrix} r_{N2} & r'_{A2} \\ r_{A2} & r'_{N2} \end{pmatrix} \tag{9}$$

As the scattering matrices are unitary, in general case they can be parametrized by four parameters each. In the situation under consideration we therefore have:

$$S_{W/2} = \begin{pmatrix} re^{i\phi_{N1}} & \sqrt{1-r^2}e^{i\phi'_{A1}} \\ \sqrt{1-r^2}e^{i\phi_{A1}} & -re^{i(\phi_{A1}+\phi'_{A1}-\phi_{N1})} \end{pmatrix} \qquad S_{-W/2} = \begin{pmatrix} re^{i\phi_{N2}} & \sqrt{1-r^2}e^{i\phi'_{A2}} \\ \sqrt{1-r^2}e^{i\phi_{A2}} & -re^{i(\phi_{A2}+\phi'_{A2}-\phi_{N2})} \end{pmatrix} \tag{10}$$

For $\epsilon = 0$ and $k_x = 0$ the scattering matrices simplify greatly as $r = 0$ (no normal reflection due to spin-momentum locking of Dirac surface states) and $\phi_{A2} = -\phi'_{A2} = -\phi_{A1} = \phi'_{A1} = \phi_A$ and we obtain Eq.(4) of the main text. In a more general scenario of $\epsilon \neq 0$, while we still have $r = 0$, there will be two Andreev reflection phases describing the scattering. The bound state equation (5) of the main text will then reduce to:

$$r_A^2 = e^{2i\frac{k_F}{k_0}\sqrt{(\epsilon-B_x)^2-\Delta^2}W}, \qquad r'^2_A = e^{-2i\frac{k_F}{k_0}\sqrt{(\epsilon+B_x)^2-\Delta^2}W} \tag{11}$$

To obtain an approximate analytical solution to these equations, we expand to linear order in ϵ both the reflection coefficients r_A and r'_A obtained from Supplementary Eq. (8) and the expression in the exponent. Then we can make use of the Lambert $\mathcal{W}$ function definition, arriving at:

$$E_\pm = \pm\Delta\,{\rm Im}\left[\frac{\sqrt{\tilde{B}_x^2-1}}{2\tilde{B}_x}\left(1 - \frac{\xi}{W}\mathcal{W}_0\left(\frac{W}{\xi}\frac{2-\tilde{B}_x^2+2i\sqrt{\tilde{B}_x^2-1}}{\tilde{B}_x^2}e^{\frac{W}{\xi}(1+2i\sqrt{\tilde{B}_x^2-1})}\right)\right)\right] \tag{12}$$

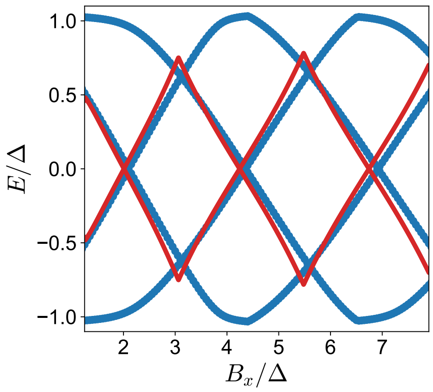

Supplementary Figure 1: Bound state energies at $k_x = 0$ for increasing Zeeman energy. Red lines show the analytical solution of Eq. (12) and blue points show the numerical results based on the tight-binding model.

where $\mathcal{W}_0(x)$ is the principal branch of the Lambert $\mathcal{W}$ function, $\tilde{B}_x = B_x/\Delta$ and $\xi = v/\Delta$. The comparison of the analytical solution with the numerical calculation based on the tight-binding model is shown in Supplementary Figure 1. The small difference in the quasiparticle branch crossing points can be attributed to the imperfect approximation of the rotationally symmetric Dirac cone in the numerical tight-binding model and the use of projected low-energy Hamiltonian in the analytical derivation.
